# Extreme Glacial Legacies: A Synthesis of the Antarctic Springtail Phylogeographic Record

**DOI:** 10.3390/insects2020062

**Published:** 2011-04-06

**Authors:** Angela McGaughran, Mark I. Stevens, Ian D. Hogg, Antonio Carapelli

**Affiliations:** 1 Max Planck Institute for Developmental Biology, Department for Evolutionary Biology, Spemannstr. 37-39/IV, Tübingen, D-72076, Germany; E-Mail: ang.mcgaughran@gmail.com; 2 South Australian Museum, and School of Earth and Environmental Sciences, University of Adelaide, SA 5000, Adelaide, Australia; 3 Centre for Biodiversity and Ecology Research, University of Waikato, Hamilton, New Zealand; E-Mail: hogg@waikato.ac.nz; 4 Department of Evolutionary Biology, University of Siena, via A. Moro 2, 53100, Siena, Italy; E-Mail: carapelli@unisi.it

**Keywords:** Antarctica, Collembola, invertebrates, glacial refugia, isolation, phylogeographic structure

## Abstract

We review current phylogeographic knowledge from across the Antarctic terrestrial landscape with a focus on springtail taxa. We describe consistent patterns of high genetic diversity and structure among populations which have persisted in glacial refugia across Antarctica over both short (<2 Mya) and long (>10 Mya) timescales. Despite a general concordance of results among species, we explain why location is important in determining population genetic patterns within bioregions. We complete our review by drawing attention to the main limitations in the field of Antarctic phylogeography, namely that the scope of geographic focus is often lacking within studies, and that large gaps remain in our phylogeographic knowledge for most terrestrial groups.

## 1. Introduction

Phylogeography is summarily about interpreting the geographic distributions of genealogical lineages. Such interpretation involves decoding the spatial and temporal components of population genetic structure from the perspective of the evolutionary and/or ecological forces that underlie it [1]. The application of metapopulation theory has been critical for integrating and interpreting molecular variation among spatially separated populations. Phylogeographic studies may aim to quantify the geographic structure of genetic variation (*i.e.*, haplotype distribution and diversity), determine the approximate timing of origin for observed structure (e.g., estimate ages of divergence) and evaluate the presence and/or direction of historical refugia and gene flow/colonization events. With appropriate sampling of genes and individuals, phylogeographers can test biogeographic hypotheses, infer processes underlying the origin, distribution and maintenance of biodiversity, and evaluate a population's demographic history [1,2]. From a conservation perspective, such analyses can prove invaluable for defining conservation units for species management purposes (e.g., [3]).

Since the pioneering work of Avise *et al.* [2], the field of phylogeography has ‘burgeoned’ [4]. Over the last three decades, identification of strong regional patterns has been used to indicate major biogeographic barriers and infer the processes that have shaped species distributions at a global scale (e.g., [5]). In particular, phylogeographic studies have identified isolating mechanisms responsible for allopatric speciation, range shifts, speciation events, the location of Pleistocene refugia, and post-glacial dispersal ‘corridors’ and barriers [6]. Much of what we know has come from a high number of studies focused on Northern Hemisphere study systems in Europe and North America (e.g., [1,7]). By contrast, the Southern Hemisphere has been less studied and phylogeographic work on Antarctic taxa is generally rare [1].

This is a special case of ‘missing the boat’ because terrestrial Antarctica is fascinating for phylogeographic study for several reasons: (1) it is a continental landmass well separated from other geographic elements [8,9]; (2) its biota is largely distinct and endemic [10–12]; (3) its glacial history is dynamic and well-studied (e.g., [13])—caveats in the accuracy of glacial reconstructions notwithstanding; and (4) there is a strong history of terrestrial Antarctic ecological research [14–17]. Therefore clear opportunities are present to test biogeographic hypotheses in terrestrial Antarctica, locate important refugial sites, provide constraints for glacial models [18,19], and by extension, help to answer biogeographically-relevant questions in other fields.

While Antarctic terrestrial ecosystems are home to the same forces that influence evolution elsewhere, the importance of isolation across both time and space is clearly discernable across the southern polar landscape and the potential impacts of such isolation on population structure are appreciable. Over time, episodes of glacial cycling have placed restrictions on species ranges and distributions, while large, stable isolating barriers (e.g., glaciers) continue to define the distribution limits within and between species [20]. Finally, other ecologically-relevant factors, such as the availability of liquid water and ice-free soil, complete the picture so that it is perhaps not surprising that Antarctic terrestrial biodiversity is depauperate [21].

In fact, the Antarctic terrestrial fauna consists entirely of invertebrates (of which only two species are ‘higher insects’, [22]), with distributions generally limited to areas of high soil moisture and/or access to water (see [23,24]). While some Antarctic invertebrates are thought to be intrinsically poor dispersers, with their absence of wings, small body size and generally poor desiccation tolerance (e.g., [25]), others are suspected to be comparatively much better at moving around [24,26,27]. In concert with environmental effects, varying dispersal capabilities among co-distributed taxa provide an ideal platform from which to examine phylogeographic signal across isolated island-like contemporary populations [28–30]. Collectively, glaciological/ecological forces and species life history traits are therefore likely to have dominated and defined evolutionary processes in Antarctic terrestrial taxa through their isolating effects (e.g., [10,31]).

For most groups of Antarctic biota, large gaps remain in both biological and biogeographical knowledge (e.g., [26,32,33]). However, Antarctic springtails (Arthropoda: Collembola) and mites (Arthropoda: Acari) are perhaps the most widely studied in this context. This review synthesizes findings and patterns emerging from phylogeographic studies of the springtails of Antarctica, drawing reference to other (non-springtail) taxa as appropriate.

## 2. Current Phylogeographic Patterns

Today, Antarctic invertebrates are restricted to ice-free regions which are scattered across just 0.34% of the continent [34]. Over recent (<2 Mya) timescales, pockets of ice-free surface—particularly coastal and lake areas—have become available following ice-front and glacial retreat after the Last Glacial Maximum (LGM, 20 kya) [35–38]. However, it is also evident that large terrestrial regions have sustained ice-free oases over multi-million (*i.e.*, >10 Mya) year timescales (e.g., [18,39]). In practice, this allows for a process by which resident taxa could have survived over the long-term in Antarctica (26 Mya from the start of glaciation), with refugia serving as centres for preservation of biodiversity during times of glacial expansion [38–40]). A history of refugial occupation can leave key genetic signatures upon contemporary populations, such as high diversity at refugial sites and strong differentiation between geographically distant sites that have maintained populations in isolation that have undergone drift.

Current phylogeographic patterns in terrestrial Antarctica are likely to be either constrained or reinforced by: (1) the isolation of Antarctica from other southern land masses; (2) the island-like nature of current habitat accessibility on a local scale; and (3) the potential for long-term refugial-based survival of (endemic) terrestrial species. These three factors are all important, but they do not act discretely, nor do they act uniformly across the Antarctic landscape.

**Figure 1:**
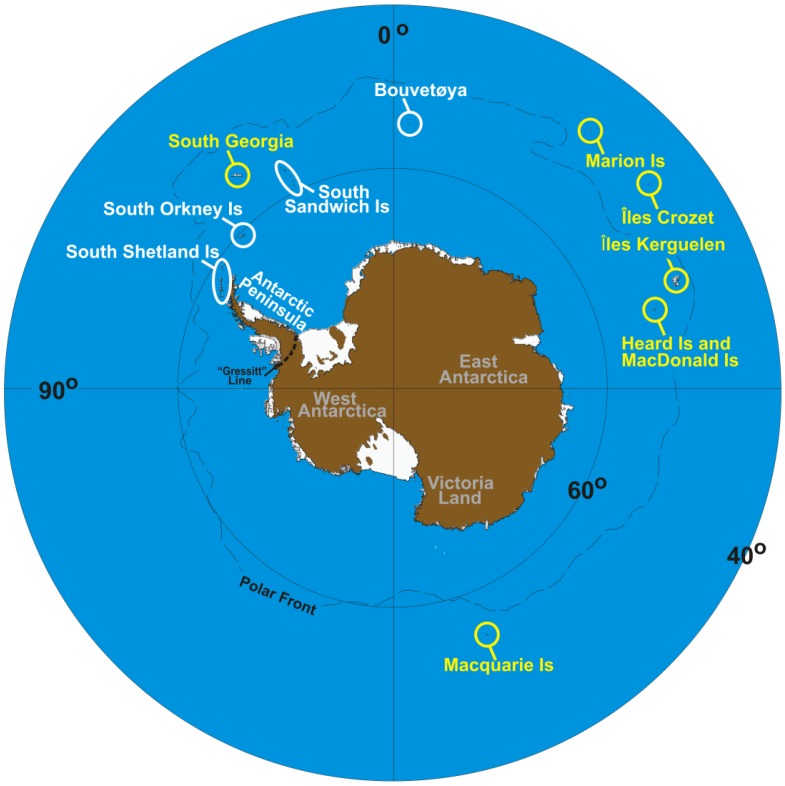
Map showing the different Antarctic zones and locations referred to in the text. Each zone is indicated by colour: yellow corresponds to sub-Antarctica, white to maritime Antarctica, and grey to continental Antarctica. In addition, the ‘Gressitt Line’ is shown at the base of the Antarctic Peninsula(see text).

Indeed, there are three generally accepted demarcation zones in Antarctica—discrete in the nature of their resident flora and fauna, their tectonic characteristics, their ecosystems, their biogeographic legacies and their climatic profiles [21,32,41–43]. These zones correspond to the sub-Antarctic (consisting of a ring of islands that surround the continent, including Macquarie Island, South Georgia, Marion Island, Îles Crozet, Îles Kerguelen and Heard and Macdonald Islands), the maritime Antarctic (including the South Shetland Islands, South Sandwich Islands, Bouvetøya the South Orkney Islands and the western side of the Antarctic Peninsula), and the continental Antarctic, which includes the rest of the continent [44–46] (Figure 1). As one moves from the outer (sub-Antarctic) to the inner (continental Antarctic) zones, the climate becomes drier and cooler, and the active season (*i.e.*, the period pertinent to terrestrial biota), shorter (< 1 or 2 months at inland locations/up to 12 months in the sub-Antarctic).

The evidence to support these ‘macro’-distinctions comes from such phenomena as the ‘Gressitt Line’—a divide between continental Antarctica and the Antarctic Peninsula, across which few terrestrial invertebrate fauna [32,47–49], or flora [33,50], are shared. Recent recognition of this bioregionalization implicates long-standing isolation both across and within Antarctica [21,39]. At finer (‘micro’) scales, separate areas within all three biogeographic zones possess high levels of regional endemism, suggesting that they too should be considered discrete [40,49–51].

Throughout each of these zones, springtail diversity is relatively well known, and up to 25 species of Collembola have been described [52]. However, just seven species have been the focus of phylogeographic studies (largely due to logistical and financial constraints). This includes three continental Antarctic species, one continental/maritime Antarctic species, and three species from the maritime Antarctic.

For most of the studies reviewed here, inferences about species history have been largely based on observed patterns of genetic variation in its geographical context. Thus, many of the general patterns we highlight stem largely from haplotype distribution analysis (e.g., haplotype networks, haplotype and nucleotide diversity indices). Where these analyses have extended to calculation of divergence times based solely on genetic distance amongst identified haplotypes, these have been based on either uncorrected or corrected (following model selection based on recommendations of suitable software) p-distances and employment of a molecular clock. In all cases, the clock used has been based on a strict molecular clock conservatively calibrated for arthropods and derived from comparisons between geological and molecular data [53,54]. To provide the most conservative estimates and to allow for differences among studies between the types of distance measure used, all of these studies have employed both the upper and lower limits of the arthropod clock (corresponding to divergence rates of 1.5 and 2.3% per million years, respectively). In addition, some of the work reviewed here employs other statistically rigorous phylogeographic approaches [4] that incorporate maximum-likelihood or Bayesian frameworks to tests of a demographic nature. In these latter cases, analyses were performed both with and without molecular clocks as required—when molecular clocks were employed, they utilized the same approach outlined above. We refer the reader in all cases to the original publications for further information regarding analyses employed and their relevant caveats.

The following sections will focus on each of the three discrete Antarctic regions separately, referring to non-springtail taxa where phylogeographic data for them exists.

### 2.1. Continental Antarctica

**Figure 2:**
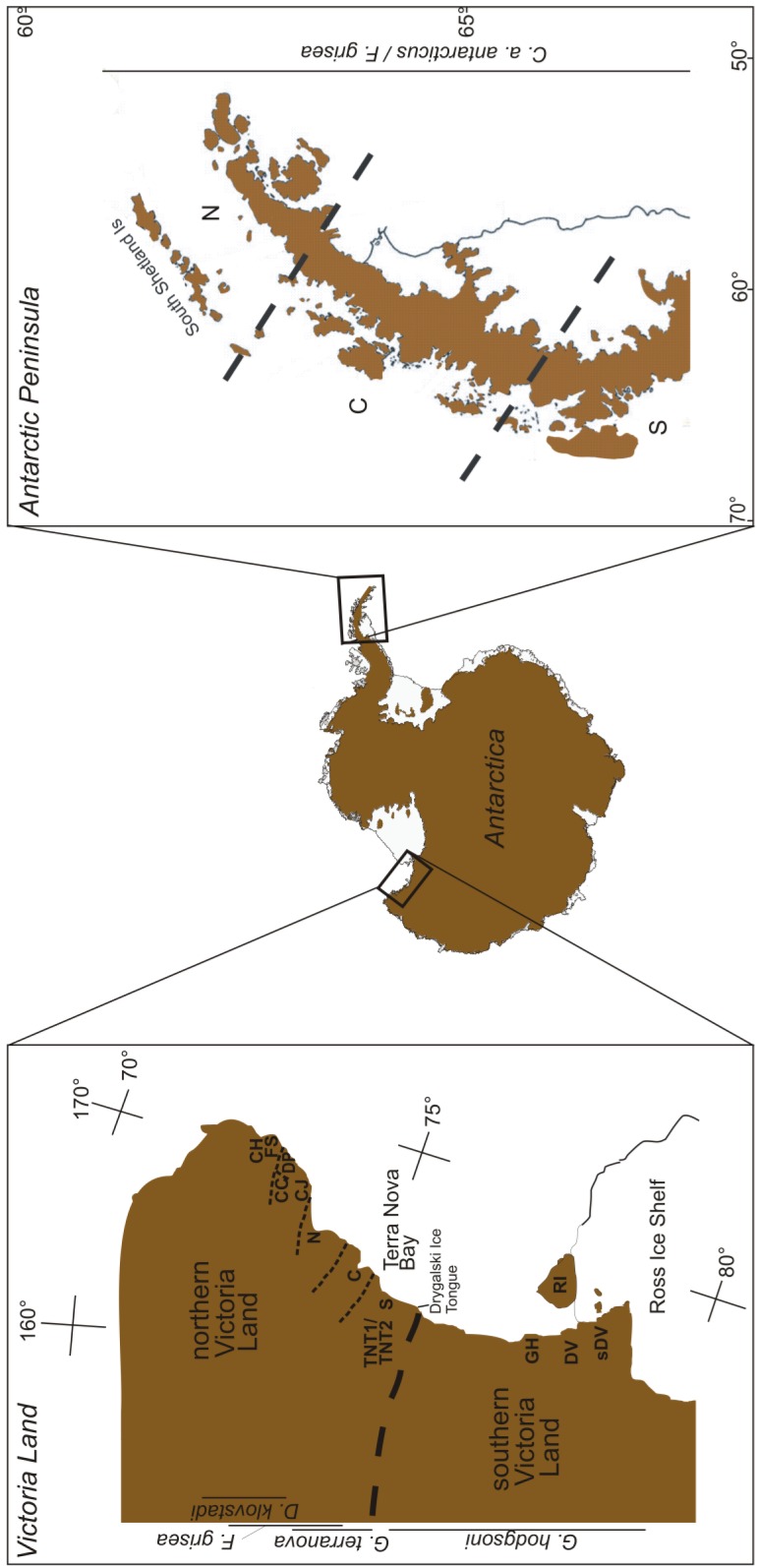
Figure showing the phylogeographic sampling that has been achieved for four Antarctic springtail species across their distributional ranges (as indicated by vertical lines beneath/above species names). The codes used in the two inset boxes are location codes and refer to potential refugial locations for each species as referred to in the respective publications and Table 1. Dashed line in the inset boxes indicate heavy or fine biogeographic breaks among regions and in many cases also represent major glacial systems. See text for further details.

Continental Antarctica is a cold, polar desert [55], home to a few vast ice-free areas, the largest of which—the McMurdo Dry Valleys of southern Victoria Land—occupies around 40,000 km^2^ [21]. Much of the phylogeographic work on the continent has centered on Victoria Land (Figure 2), and will subsequently form the bulk of our focus here.

Springtails are found throughout Victoria Land (Figure 1), where the distribution of individual species is generally constrained by distinct biogeographic breaks [26,56]. The northern and southern parts of the region are separated by the Drygalski ice tongue (Figure 2), which appears responsible for maintaining isolation between species in the two regions [26,56]. Northern Victoria Land has four springtail species: *Desoria klovstadi* (Carpenter), *Gressittacantha terranova* Wise, *Friesea grisea* Schäffer, and *Cryptopygus cisantarcticus* Wise [26]. Of these, the former two have largely non-overlapping distributions that are heavily partitioned by the presence of glaciers, while the distributions of the latter two species are mostly sympatric and scattered across northern Victoria Land. Southern Victoria Land has three species of springtail, the most widely distributed of which is *Gomphiocephalus hodgsoni* Carpenter [10,57,58]. Two other species of springtail (*Neocryptopygus nivicolus* Salmon and *Antarcticinella monoculata* Salmon) have more restricted distributions in the region and have been found at a few sites near Granite Harbour (GH; Figure 2) [12,59–61]. Of the seven springtails in Victoria Land, four have been examined phylogeographically. This includes *D. klovstadi* [31,61], *Gr. terranova* [62,63] and *F. grisea* [64] from northern Victoria Land, and *Go. hodgsoni* from southern Victoria Land [10,55].

For *D. klovstadi*, cytochrome *c* oxidase subunit II (COII) analysis focused on 69 individuals from five geographic locations (Figure 2; Table 1). Divergence among 26 identified haplotypes ranged up to 1.6%, placing the genealogical history of the populations for this species potentially within the last million years [61]. This analysis revealed striking population structure associated with geography, with just one instance of haplotype sharing among locations (between locations Football Saddle (FS) and Cape Jones (CJ); see Figure 2) [61]. This degree of segregation suggests a history of isolation between the locations while the presence of higher diversity at two of the sites (Daniell Peninsula (DP) and Cape Hallett (CH); Figure 2), highlights them as potential refugia from which the other lower diversity satellite populations may have been founded in more recent times. If this is the case, it is unusual that there are no remaining haplotype links between the two source populations and their ‘sinks’. Hence, it is also possible that each of the five populations are in fact refugial remnants or are the result of independent founding from as yet unsampled refugia. It would be particularly interesting to see if such heterogeneity among populations is a common feature throughout the northern area of the distribution range of *D. klovstadi* that is currently unsampled (see Figure 1 in [61]).

From the southern parts of northern Victoria Land, *Gr. terranova* has been examined using both allozymes [60] and mtDNA cytochrome *c* oxidase subunit I (COI) gene [63] (Table 1). Fanciulli *et al.* sampled 22 populations across the distributional range of *Gr. terranova* and found high levels of allelic variability among populations, even those in close geographical proximity [62]. These authors also found that populations could be arranged into three groups among which little gene flow was inferred from allelic frequencies. These groups corresponded to northern, central and southern parts of the distributional range, and were bounded in each case by major glacial systems (see Figure 2). While no time frame was provided by Fanciulli *et al.* [62], the high degree of differentiation among populations was taken as a possible indicator of ancient divergence for this species. Hawes *et al.* took an mtDNA (COI) approach, examining *Gr. terranova* samples taken from two altitudinal transects and a third more distant population in the Terra Nova Bay region (Figure 2) [63]. Here, the authors again found high genetic variability among samples despite close geographic proximity, with an upper divergence of 10% (*i.e.*, corresponding to Pliocene divergence around 4-5 Mya) separating two distinct haplotype groups [63]. This suggests that *Gr. terranova* may be undergoing sympatric speciation, as has been suggested previously for *Go. hodgsoni* (e.g., [57]). Alternatively, two major genetic clades that were previously geographically isolated may have undergone recent (and now over-lapping) expansion from glacial refugia. Hawes *et al.* also found that, while *Gr. terranova* haplotypes showed little geographic structuring, sites with freshwater bodies seemed to be centers of haplotype diversity and, in several cases, links between geographically-isolated populations seemed to be accounted for by stochastic colonization events [63].

Finally, Torricelli *et al.* [64] examined *F. griesea* from northern Victoria Land using two mtDNA genes (ATP6 and COI). These authors found a total of 10 haplotypes among 55 individuals, which were separated by 10.9% COI sequence divergence (*i.e.*, pre-Pleistocene origin) (Table 1). This high divergence resulted from a distinct group of Cape Hallett (CH; Figure 2) individuals. Within the southern portion of their sampling range, divergence among haplotypes was lower (<2.9%), although a population at Crater Cirque (CC; Figure 2) also remained distinct from the other populations [64]. The differentiation between northern and southern parts of the continental range of this species is in agreement with findings for other springtails in the region [61–63]. This suggests that the major glacial systems in the area have strongly impacted contemporary population structure by maintaining isolation among all refugial populations even as conditions have ameliorated.

In southern Victoria Land, *Go. hodgsoni* is arguably the most extensively studied Antarctic springtail in phylogeographic [10,49,57,65,66], ecophysiological (e.g., [67–69]) and now phylogenetic [58] contexts. This species is widespread throughout southern Victoria Land (Figure 2) and has been examined from both macro- and micro-scales across its range using both allozymes and mtDNA (COI and COII) (Figure 2, Table 1). In the most comprehensive analysis to date, McGaughran *et al.* sampled 289 and 191 *Go. hodgsoni* individuals for COI and COII, respectively, employing both descriptive haplotype distribution and diversity index analyses as well as nested cladistic analysis and a variety of demographic approaches (including maximum-likelihood based analyses) [49]. The demographic analyses in this study (using the programs ARLEQUIN, FLUCTUATE and MDIV; see [49]) all placed the genealogical history of *Go. hodgsoni* into timescales relevant to the last million years. In addition, haplotype distribution analysis showed an overall population structure for *Go. hodgsoni* that was compatible with the presence of historical glacial refugia maintaining isolated populations over this time. In essence, this resulted in a very fragmented haplotype pattern, with several putatively ancestral haplotypes present in high frequency. These haplotypes were rarely shared among populations for both COI (*ca.* 29%) and COII (21%) and most sharing was within large proposed refugial regions. However, there were a few isolated instances of haplotype sharing among regions (e.g., the McMurdo Dry Valleys (DV, sDV), Granite Harbour (GH); Figure 2). This suggests that isolated, stochastic, often long-distance colonization events are responsible for maintaining links across the gene pool for this species, and that occasional founding events have successfully established new populations (e.g., on Ross Island (RI); Figure 2) [49].

In southern Victoria Land, genetic structure has also been examined for the endemic mite, *Stereotydeus mollis* Strandtmann, which has an almost identical geographic distribution to *Go. hodgsoni* and often coexists in the same location [60,66]. In general, population genetic patterns of *S. mollis* have been shown to be congruent with those demonstrated for *Go. hodgsoni* and it is clear that *S. mollis* populations have also been dominated by contraction into refugia during glacial expansions [10,66]. In comparison to *Go. hodgsoni* however, *S. mollis* has shown far greater levels of COI sequence divergence (up to 14.4%, compared to 2.1% for *G. hodgsoni*) and therefore, further work is needed to clarify taxonomic issues for this species [66,70–73].

Another non-springtail study for comparison focused on the nematode *Scottnema lindsayae* Timm, which is found throughout southern Victoria Land as well as in regions to the north and south of the Transantarctic Mountains. In this species, no evidence has been found for geographical structuring of either mtDNA, 28s ribosomal DNA (rDNA) or ribosomal RNA (rRNA) haplotypes [27,74], and it has been suggested that the comparative ease of dispersal in nematodes prevents heterogeneity among populations from establishing [27,74,75]. However, though highly capable of long-distance dispersal, Antarctic nematode fauna are almost entirely endemic [26,76], suggesting that, while dispersal within Antarctica may be prevalent, dispersal into Antarctica offers more significant barriers to success.

In contrast, the moss *Sarconeurum glaciale* Card. & Bryhn, was found to have patterns of genetic differentiation (RAPD's) between Taylor Valley and Ross Island populations (DV and RI, respectively; Figure 2), while within Taylor Valley, the same authors detected high genetic (RAPD) variability between two isolated populations of the moss *Bryum pseudotriquetrum* (Hedw.) [77]. In the case of mosses, asexual reproduction coupled with limited propagule dispersal likely enhances genetic structuring. More recent work examining nuclear rRNA loci (18S–26S (ITS) [78], and ITS+*phy2* [50]) of continental Antarctic populations of the moss *Bryum argenteum* Hedw., highlighted this species' isolation in Victoria Land (compared to sub-Antarctic and temperate populations). It also supported a disjunction between southern and northern locations within Victoria Land [50,78], which is very similar to the genetic breaks described above for springtails in the same region (e.g., [61]).

Thus, throughout continental Antarctica, common patterns within biogeographically distinct sub-regions may reflect a common history of isolation, persistence over Mya timescales, and re-colonization for terrestrial flora and fauna. Studies in the region have detected spatial genetic structure over both small (<1 km) and large (hundreds of kms) geographic distances (e.g., [10,49,61]) that span either: (1) Mio-Pliocene (4 – 5 Ma; *Gr. terranova*), or (2) Pleistocene (< 2 Ma; *D. klovstadi* and *Go. hodgsoni*) timescales, and recent work suggests that closer examination may reveal similar long-term isolation and population sub-structuring for other Antarctic biota (e.g., [50]). These data indicate minimal or no present-day gene flow among populations of the same putative species, despite their sometimes close geographic proximity. Thus, while re-colonization from refugia has played a significant role in determining contemporary patterns of population structure throughout continental Antarctica, this is likely to have taken the form of rare stochastic dispersal events. Such stochastic colonization processes are also likely to have largely driven species distributions in other Antarctic taxa (e.g., [79,80]), although these have not yet been the focus of studies employing molecular analyses.

### 2.2. Maritime Antarctic

In contrast to continental Antarctica, with its large expanse of rocks and well-developed coastal and inland terrestrial ecosystems, the maritime Antarctic consists of several small islands, and the western half of the Antarctic Peninsula (Figure 1). These areas are largely covered by snow and ice and peppered with terrestrial oases that are generally restricted in location to coastal areas [21]. Here, the springtail *Cryptopygus antarcticus* is the most widespread and abundant terrestrial arthropod [22].

The subspecies *C. antarcticus antarcticus* Willem, has been examined using mtDNA (COI and COII) sequences for 379 individuals across 24 locations throughout the Antarctic Peninsula and associated South Shetland Island archipelago [49]. Haplotype distribution analysis has found strong evidence of population structure and high levels of genetic diversity among locations [49]. Collapsing these locations into Northern, Central and Southern regions found little evidence of population linkage, with haplotypes shared in just a few instances, most of which were within these regions. There was also a striking excess of haplotypes present in low frequency, particularly in the mtDNA COI dataset (Table 1), suggesting recent population expansion for this species that has nearly obliterated ancestral signal [49].

To put their results into a greater biogeographic context, McGaughran *et al.* compared the patterns found for *C. a. antarcticus* on the Peninsula to findings for *Go. hodgsoni* on the continent (see previous section) [49]. In particular, these authors were interested in whether the two species, whose life history strategies and evolutionary persistence in Antarctica are postulated to be similar, would show similar contemporary patterns of population structure [48]. This theory was tested using demographic analyses (e.g., in the programs ARLEQUIN, FLUCTUATE and MDIV) to complement haplotype distribution analysis. With this approach, the strong genetic subdivision found in both species was shown to support a pattern in which multiple glacial refugia have allowed post-glacial expansion during the Pleistocene (including the last LGM), and subsequent population growth in isolation. However, there were distinct, and informative, differences in the finer scale of these patterns. For example, while both species showed evidence for population expansion, in *C. a. antarcticus* this appears to have been of a much greater magnitude and suggests that successful founding events on the Peninsula have been much more frequent through time [49].

In a more recent study, Torricelli *et al.* examined *F. grisea* from locations in continental Antarctica (see above) and the Antarctic Peninsula [64]. From a mtDNA (ATP6/COI) dataset of 80 individuals and nine populations, these authors obtained seven Peninsula haplotypes (<2.7% divergent) that differentiated into unique northern and southern groups. When compared to the Victoria Land dataset, COI divergence was 21.3% at its greatest, thus the taxonomic status of *F. grisea* has been questioned [64]. Indeed, work based on complete mitochondrial DNA sequences has shown that rather than being one widespread species, *F. grisea* is best described as separate species in their respective disparate locations [47], and this has recently been confirmed using morphological characters and nuclear DNA sequences [48]. The sequence divergence observed here corresponds to a speciation event initiated well into the Miocene [47], and is consistent with the hypothesis that Victoria Land (and continental Antarctica in general), has been isolated since that time [11].

The distinct intra-regional northern and southern genetic breaks found in *F. grisea* mirror the findings outlined above for *C. a. antarcticus* and *Go. hodgsoni*. Thus, while life in different areas of Antarctica is likely to be similar in certain respects, the different regions have imposed different genetic legacies upon their inhabitants, and this is consistent with the evidence outlined above supporting both macro- and micro-distinctions among the different Antarctic biogeographic elements (e.g., [21,32,33,40,47,49,51,69]). An interesting distinction between population bottlenecks/expansions of benthic relative to pelagic Antarctic shelf fish [81] shows that such differentiation among regions may not be restricted to the terrestrial realm.

Finally, in a non-springtail example, the southern maritime Antarctic hairgrass *Deschampsia antarctica* Desv. provides yet another illustration of isolation of populations among regions. This species showed overall low genetic diversity in an AFLP analysis. However, no genotypes were shared among populations from Signy Island to the north and from populations further south (Figure 2). Even within these two areas, significant genetic differentiation was found to exist between subpopulations separated by as little as 4 km [82]. Other differences in the fauna of the northern and southern parts of the maritime Antarctic have also been observed [20,83].

Thus, the pattern of isolation among subpopulations and refugial legacies prevalent in the continental Antarctic is recapitulated in maritime Antarctica. Because much of the presently available habitat in maritime areas is suggested to have been generally inundated by ice sheets at the LGM [84], many refugial locations have been difficult to identify [85]. However, pre-LGM refugia are beginning to be described [49,76] and these are likely to have been extremely important in creating maritime Antarctica's unique contemporary terrestrial fauna.

### 2.3. Sub-Antarctic

In comparison to the continental and maritime Antarctic, some islands of the sub-Antarctic have been well characterized (e.g., [86,87]). One interesting feature is that the circum-polar islands, of different age and in some cases, different geology, show an east-west division in species distribution [83]. Thus, species in the eastern parts of the zone (e.g., Macquarie Island and Îles Crozet) generally do not occur in the western (e.g., South Georgia) parts of the zone, and vice versa (Figure 2). Like the rest of Antarctica, the sub-Antarctic has a history of landscape subdivision through glaciation and/or volcanic activity [88–93], and indigenous fauna have histories extending in some cases to multi-million year timescales [11,32,94,95].

All of the sub-Antarctic islands are characterized by two major biotopes [96]. These correspond generally to an older epilithic biotope and a younger vegetated biotope dominated by eurytopic colonists [96,97]. Most sub-Antarctic islands are therefore inhabited by a mixture of ancient native taxa and young invading species that have successfully established, particularly following human exploration [32]. This highlights a key difference between the sub-Antarctic and other Antarctic zones: immigration into (and within) the sub-Antarctic is a much more common occurrence than at other less penetrable maritime and continental sites. Thus, many elements of the sub-Antarctic fauna are recent immigrants, originating either from temperate locations outside the polar frontal zone, or from the maritime Antarctic [32,98]. Such colonization in the case of springtails is likely to have been aided by their ability to raft for weeks at a time on both sea and fresh water [15,99,100], and may have been aided in some part by the ‘west wind drift’ and other oceanic currents as new island habitats have become available (e.g., [95]). The mixture of young and old taxa in the sub-Antarctic is particularly exemplified by springtails, which have been shown to have a varied and diverse evolutionary history in the circum-Antarctic that consists of both ancient and recent elements [11,95].

At island-scales, the mix of ancient and recent species is expected to be reflected in a pattern of substantially greater population structure in indigenous species (due their survival in isolated refugia over time), compared to introduced species whose residency time has been shorter. One study employing haplotype distribution analysis looked at this in detail and found exactly this pattern [96]. Myburgh *et al.* examined mtDNA (COI) genetic substructure in two indigenous and two introduced springtails on Marion Island [96]. They found considerable genetic structure for the indigenous species (e.g., 35 and 13 haplotypes from the springtails *Cryptopygus antarcticus travei* Deharveng and *Tullbergia bisetosa* Börner, respectively) and no structure (*i.e.*, a single haplotype) in both of the introduced species (Table 1). For the two native species, the genetic patterns were also compatible with the geological and glaciological history of the island [96].

This result was supported by a second haplotype distribution analysis of *C. a. travei* from Marion Island [101]. This work used a dataset containing 113 individuals to obtain 39 unique COI haplotypes, of which 30 were singletons. This high degree of genetic diversity and haplotype distribution among populations supports long-term isolation and limited gene flow in *C. a. travei* [101]. Analysis of this species [101], and also of the mite *Eupodes minutus* [102] supported a north-east/south-west divide among the Marion Island populations that also corresponded to life history traits (e.g., patterns of population metabolic rate structure) in the case of *C. a. travei* [101]. This finding of geographical demarcation is consistent with the occurrence of multiple volcanic events on the southern side of Marion Island in conjunction with glaciation events over time.

Thus, a diverse genealogical history impacted strongly upon by events resulting in isolation of populations in refugia, is a pattern not only common to the deeper continental Antarctic, but also prevalent at its most northern (sub-Antarctic) locations.

## 3. Conclusions

While a large proportion of Antarctic terrestrial biota remains understudied [21,26,32,33,103], and the work on springtails has only scratched the tip of the phylogeographic iceberg, at least four main themes have emerged.

First, Antarctic springtail populations, regardless of their zonal location, are characterized by high genetic diversity and strong genetic structure. This fragmentation is represented by the presence of unique haplotypes within populations and a very low degree of haplotype sharing between populations. It is generally maintained by the isolating nature of Antarctic terrestrial habitats—each population is an island—over both contemporary and historical timescales. In some cases, this isolation has led to incipient (e.g., [57,62–64]) or ancient (e.g., [11,47]) speciation. Second, over historical timescales, springtail populations have persisted in Antarctica in refugial sites which have remained ice-free, in some cases, for millions of years. Refugia enabling the long-term persistence of Antarctic taxa have resulted in a dynamic contemporary fauna consisting of both recent (<2 Mya) and ancient (>10 Mya) elements. Third, the presence of a small number of shared haplotypes between non-contiguous sites suggests occasional (and successful) long-distance dispersal events from these refugia. This has enabled the establishment of satellite populations and the maintenance of links between these and their ancestral populations. Fourth, location matters. Although phylogeographic patterns amongst Antarctica's bioregions are generally concordant, differences among sites in factors such as dispersal opportunities, local climate and length of active season, are reflected in divergent demographic and population genetic signatures among species in different locations. This last point emphasizes the lack of representativeness of any one population or region of Antarctic biota, and should be taken into account when developing conservation strategies, and especially in light of future climate changes.

## 4. Future Directions

**Figure 3:**
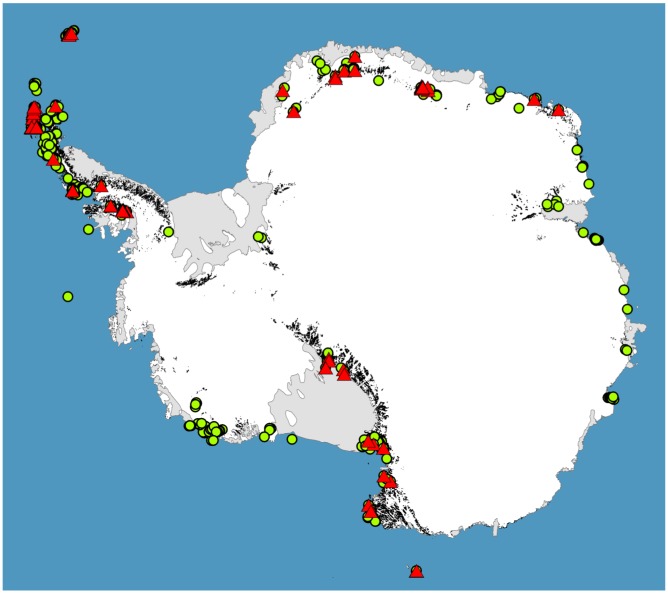
ArcGIS Map of terrestrial invertebrate occurrence records and ice-free terrain [104,105]. Areas shaded in black represent the ice-free terrain where no terrestrial invertebrate collections have been made; red triangles illustrate all springtail records while green circles illustrate all other terrestrial invertebrate records.

For much of the Antarctic biota, survey data is limiting or completely lacking ([26,32,33], see Figure 3), and as we have shown here, large gaps remain in our phylogeographic knowledge for most terrestrial groups. However, accurate phylogeographic hypotheses require sampling across entire species ranges. While sampling effort in Antarctica has been greatest for the microarthropods [26], the springtail *Go. hodgsoni* from continental southern Victoria Land is the only species for which we can reliably say this level of sampling has been achieved ([49], and references therein). Thus, the underlying future directive of Antarctic phylogeography must be to sample species across as wider proportion of their geographic range as possible. In addition, some large important biogeographic ice-free areas remain unsampled for any species (see Figure 3). Thus, there are currently no data from inland locations of the Antarctic Peninsula, including several areas thought to be most similar to the outer regions of continental Antarctica [51]. Sampling of these and other ice-free locations (e.g., [19,32]) would be beneficial for further assessment of how regional differences may have influenced distinct evolutionary histories in local taxa.

In addition to ‘more locations’, we can give more confidence to studies showing concordant patterns across taxa as this often reveals a concerted response of species and communities to the historical environmental changes that have shaped their present distributions. Hence, ‘more species’ are a second required future directive.

Finally, analysis of ‘more genes’, especially combining the use of both nuclear and mitochondrial sequence data, is an optimal future strategy for phylogeographic studies. This approach can provide information over different temporal scales, accommodate coalescent stochasticity and improve the accuracy of inferences about past demography and divergence times [4,106–109]. We note this particularly as our review has highlighted a possible trend towards the absence of rigorous statistical phylogeographic analyses in current studies of Antarctic biota. Such analyses would enable tests of hypotheses about migration, growth rates, mutations rates, population structure, and population divergence within a modeling framework. However, much of the software currently available to service this approach requires multilocus datasets (see Table 4 in [4]). Future employment of such modeling-based analyses will also be particularly important for detecting the influence of haplotype extinctions and lineage sorting (see e.g., [1,49,109]). Notwithstanding multi-locus approaches, is the potential for second and/or third generation sequencing to address phylogeographic questions from a ‘population genomics’ perspective.

While this review highlights the ‘work in progress’ nature of the field currently (with this largely due to the logistical constraints of Antarctic fieldwork), the promise of such future approaches shows that the outlook for Antarctic phylogeography is an exciting one. There is much to be gained from Antarctic studies—particularly how populations have responded and may respond to long-term environmental changes. Previous phylogeographic research, reviewed here, will provide a valuable platform from which to expand this knowledge in the years to come.

## Figures and Tables

**Table 1:** Summary of phylogeographic analyses performed to date on springtails in Antarctica, including information on species, location, gene (all mitochondrial DNA), number of individuals sampled, (N); number of haplotypes; N_h_; mean number of haplotypes per location/population, (H_loc_); percentage of haplotypes shared among locations/populations, (H_sha_); percentage divergence (uncorrected p-distance) range among haplotypes (H_div_); phylogeographically-proposed refugial location(s); and reference to the literature. nVL and sVL: northern and southern Victoria Land, respectively. See Figure 1 for proposed refugial location codes.

**Species**	**Location**	**Gene**	**N**	**N_h_**	**H_loc_**	**H_sha_**	**H_div_**	Proposed Refugia*	**Reference**
*Desoria klovstadi*	nVL	COII	69	26	5.4	4	0.1–1.6	DP, CH, CC, FS, CJ	[61]^1^
*Gressittacantha terranova*	nVL	COI	54	26	2.7	4	0.2–10.4	TNT1, TNT2 N, C, S^2^	[63]^2^
*Gomphiocephalus hodgsoni*	sVL	COI	289	45	3.2	29	0.2–2.5	GH, DV, sDV, RI	[49]^3^
		COII	191	58	4.2	21	0–2.9	GH, DV, sDV, RI	
*Friesea grisea*	nVL	COI	55	10	1.3	10	0–10.9	CH, CC	[64]
	Antarctic Peninsula	COI	80	7	0.8	29	0–2.7	N, S	[64]
*Cryptopygus antarcticus antarcticus*	Antarctic Peninsula	COI	139	89	6.9	11	0–9.2	N, C, S	[49]^3^
		COII	240	73	4.6	19	0–3.3	N, C, S	
*Cryptopygus antarcticus travei*	Marion Island	COI	113	39	4.9	15	0–2.9	Katedraalkrans^4^	[101]^4^
*Tullbergia bisetosa*	Marion Island	COI	40	13	2.8	31	0–1.7	n/a	[96]

* See Figure 2;

1 See also [31];

2 See also [61];

3 See also [10,56,64,65];

4 See also [95]

## References

[1] Beheregaray L.B. (2008). Twenty years of phylogeography: The state of the field and the challenges for the Southern Hemisphere. Mol. Ecol..

[2] Avise J.C., Lansman R.A., Shade R.O. (1979). The use of restriction endonucleases to measure mitochondrial DNA sequence relatedness in natural populations. I. Population structure and evolution in the genus *Peromyscus*. Genetics.

[3] Moritz C. (1994). Defining ‘evolutionary significant units’ for conservation. Trends Ecol. Evol..

[4] Knowles L.L. (2004). The burgeoning field of statistical phylogeography. J. Evol. Biol..

[5] Soltis D.E., Morris A.B., McLachlan J.S., Manos P.S., Soltis P.S. (2006). Comparative phylogeography of unglaciated eastern North America. Mol. Ecol..

[6] Parker K., Markwith S. (2007). Expanding biogeographic horizons with genetic approaches. Geogr. Compass.

[7] Hewitt G. (2000). The genetic legacy of the Quaternary ice ages. Nature.

[8] Clarke A., Barnes D.K.A., Hodgson D.A. (2005). How isolated is Antarctica?. Trends Ecol. Evol..

[9] Barnes D.K.A., Hodgson D.A., Convey P., Allen C., Clarke A. (2006). Incursion and excursion of Antarctic biota: past, present and future. Global Ecol. Biogeogr..

[10] McGaughran A., Hogg I.D., Stevens M.I. (2008). Patterns of population structure for springtails and mites in southern Victoria Land, Antarctica. Mol. Phylogen. Evol..

[11] Stevens M.I., Greenslade P., Hogg I.D., Sunnucks P. (2006). Examining Southern Hemisphere springtails: could any have survived glaciation of Antarctica?. Mol. Biol. Evol..

[12] Wise K.A.J. (1971). The Collembola of Antarctica. Pac. Insects Monogr..

[13] Denton G.H., Hughes T.J. (2000). Reconstruction of the Ross ice drainage system, Antarctica, at the last glacial maximum. Geogr. Ann..

[14] Gressitt J.L., Leech T.S., Wise K.A.J. (1963). Entomological investigations in Antarctica. Pac. Insects.

[15] Gressitt J.L., Gressitt J.L. (1967). Entomology of Antarctica. Antarctic Research Series.

[16] Wise K.A.J., Spain A.V. (1967). Entomological investigations in Antarctica 1963–64 season. Pac. Insects.

[17] Gressitt J.L. (1971). Antarctic entomology with emphasis on biogeographical aspects. Pac. Insects Monogr..

[18] Convey P., Gibson J.A.E., Hillenbrand C.-D., Hodgson D.A., Pugh P.J.A., Smellie J.L., Stevens M.I. (2008). Antarctic terrestrial life—Challenging the history of the frozen continent?. Biol. Rev..

[19] Convey P., Stevens M.I., Hodgson D.A., Smellie J.L., Hillenbrand C.-D., Barnes D.K.A., Clarke A., Pugh P.J.A., Linse K., Cary S.C. (2009). Exploring biological constraints on the glacial history of Antarctica. Quatern. Sci. Rev..

[20] Rogers A.D. (2007). Evolution and biodiversity of Antarctic organisms: A molecular perspective. Philos. T. Roy. Soc. Lond. B.

[21] Convey P. (2010). Terrestrial biodiversity in Antarctica: recent advances and future challenges. Polar Sci..

[22] Block W., Laws R.M. (1984). Terrestrial microbiology, invertebrates and ecosystem. Antarctic Ecology.

[23] Kennedy A.D. (1993). Water as a limiting factor in the Antarctic terrestrial environment—A biogeographical synthesis. Arctic Alpine Res..

[24] Hogg I.D., Stevens M.I., Beyer L., Bölter M. (2002). Soil Fauna of Antarctic Coastal Landscapes. Geoecology of Antarctic Ice-Free Coastal Landscapes, Ecological Studies Analysis and Synthesis.

[25] Convey P. (1996). The influence of environmental characteristics on life history attributes of Antarctic terrestrial biota. Biol. Rev..

[26] Adams B.J., Bardgett R.D., Ayres E., Wall D.H., Aislabie J., Bamforth S., Bargagli R., Cary C., Cavacini P., Connell L., Convey P., Fell J.W., Frati F., Hogg I.D., Newsham K.K., O'Donnell A., Russell N., Seppelt R.D., Stevens M.I. (2006). Diversity and distribution of Victoria Land biota. Soil Biol. Biochem..

[27] Adams B.J., Wall D.H., Gozel U., Dillman A.R., Chaston J.M., Hogg I.D. (2007). The southernmost worm, *Scottnema lindsayae* (Nematoda): Diversity, dispersal and ecological stability. Polar Biol..

[28] Beyer L., Bölter M., Beyer L., Bölter M. (2002). Geoecology of Antarctic Ice-Free Coastal Landscapes, Ecological Studies Analysis and Synthesis.

[29] Bergstrom D., Chown S.L. (1999). Life at the front: history, ecology and change on southern ocean islands. Trends Ecol. Evol..

[30] Bergstrom D.M., Convey P., Huiskes A.H.L., Bergstrom D.M., Convey P., Huiskes A.H.L. (2006). Trends in Antarctic Terrestrial and Limnetic Ecosystems: Antarctica as a Global Indicator.

[31] Frati F., Spinsanti G., Dallai R. (2001). Genetic variation of mtCOII gene sequences in the collembolan *Isotoma klovstadi* from Victoria Land, Antarctica: evidence for population differentiation. Polar Biol..

[32] Chown S.L., Convey P. (2007). Spatial and temporal variability across life's hierarchies in the terrestrial Antarctic. Philos. T. Roy. Soc. Lond. B.

[33] Peat H.J., Clarke A., Convey P. (2007). Diversity and biogeography of the Antarctic flora. J. Biogeog..

[34] British Antarctic Survey Antarctica, 1:10000000 scale map. BAS (Misc). http://www.antarctica.ac.uk/Resources/schoolzone/resources/Factsheets/index.html.

[35] Burgess J.S., Spate A.P., Shevlin J. (1994). The onset of deglaciation in the Larsemann Hills, Eastern Antarctica. Antarct. Sci..

[36] Gore D.B., Rhodes E.J., Augustinus P.C., Leishman M.R., Colhoun E.A., Rees-Jones J. (2001). Bunger Hill, East Antarctica: ice free at the last glacial maximum. Geology.

[37] Hodgson D.A., Noon P.E., Vyverman W., Bryant C.L., Gore D.B., Appleby P., Gilmour M., Verleyen E., Sabbe K., Jones V.J., Ellis- Evans J.C., Wood P.B. (2001). Were the Larsemann Hills ice-free through the Last Glacial Maximum?. Antarct. Sci..

[38] Cromer L., Gibson J.A.E., Swadling K.M., Hodgson D.A. (2006). Evidence for a faunal refuge in the Larsemann Hills, East Antarctica, during the Last Glacial Maximum. J. Biogeogr..

[39] Convey P., Stevens M.I. (2007). Antarctic biodiversity. Science.

[40] Vyverman W., Verleyen E., Wilmotte A., Hodgson D.A., Willem A., Peeters K., Van de Vijver B., De Wever A., Leliaert F., Sabbe K. (2010). Evidence for widespread endemism among Antarctic micro-organisms. Polar Sci..

[41] Walton D.W.H., Laws R.M. (1984). The terrestrial environment. Antarctic Ecology.

[42] Convey P. (1996). Overwintering strategies of terrestrial invertebrates in Antarctica—The significance of flexibility in extremely seasonal environments. Eur. J. Entomol..

[43] Cande S.C., Stock J.M., Müller R.D., Ishihara T. (2000). Cenozoic motion between East and West Antarctica. Nature.

[44] Lewis-Smith R.I., Laws R.M. (1984). Terrestrial plant biology of the sub-Antarctic and Antarctic. Antarctic Ecology.

[45] Longton R.E. (1998). Biology of Polar Bryophytes and Lichens.

[46] Convey P., Levin S.A. (2007). Antarctic ecosystems. Encyclopedia of Biodiversity.

[47] Torricelli G., Carapelli A., Convey P., Nardi F., Boore J.L., Frati F. (2010). High divergence across the whole mitochondrial genome in the ‘pan-Antarctic’ springtail *Friesea grisea*: Evidence for cryptic species?. Gene.

[b48-insects-02-00062] 48.Stevens, M.I.; Greenslade, P.; D'Haese, C.A.; Torricelli, G.; Convey, P.; Carapelli, A.; Frati, F. *Frisea grisea* from Victoria Land, Antarctic Peninsula, Molodezhnaya and Tereshkovoi Oasis and the type locality of South Georgia, are currently the subject of taxonomic revision. Using molecular (mitochondrial and nuclear DNA) and morphological characters, this work supports separate species among each of these locations. 2011, unpublished work.

[49] McGaughran A., Torricelli G., Carapelli A., Frati F., Stevens M.I., Convey P., Hogg I.D. (2009). Contrasting phylogeographic patterns for springtails reflect different evolutionary histories between the Antarctic Peninsula and continental Antarctica. J. Biogeogr..

[50] Hills S.F.K., Stevens M.I., Gemmill C.E.C. (2010). Molecular support for Pleistocene persistence of the continental Antarctic moss *Bryum argenteum*. Antarct. Sci..

[51] Pugh P.J.A., Convey P. (2008). Surviving out in the cold: Antarctic endemic invertebrates and their refugia. J. Biogeogr..

[52] Greenslade P. (1995). Collembola from the Scotia Arc and Antarctic Peninsula including description of two new species and notes on biogeography. Pol. Pis. Entomol..

[53] Brower A.V.Z. (1994). Rapid morphological radiation and convergence among races of the butterfly *Heliconius erato* inferred from patterns of mitochondrial DNA evolution. P. Natl. Acad. Sci. USA.

[54] Quek S.-P., Davies S.J., Itino T., Pierce N.E. (2004). Codiversification in an ant-plant mutualism: stem texture and the evolution of host use in Crematogaster (Formicidae: Myrmicinae) inhabitants of Macaranga (Euphorbiaceae). Evolution.

[55] Sømme L. (1995). Invertebrates in Hot and Cold Arid Environments.

[56] Sinclair B.J., Stevens M.I. (2006). Terrestrial microarthropods of Victoria Land and Queen Maud Mountains, Antarctica: implications of climate change. Soil Biol. Biochem..

[57] Stevens M.I., Hogg I.D. (2003). Long-term isolation and recent range-expansion from glacial refugia revealed for the endemic springtail *Gomphiocephalus hodgsoni* from Victoria Land, Antarctica. Mol. Ecol..

[58] Greenslade P., Stevens M.I., Torricelli G., D'Haese C.A. (2011). An ancient Antarctic endemic genus restored: morphological and molecular support for *Gomphiocephalus hodgsoni* (Collembola: Hypogastruridae). Syst. Entomol..

[59] Wise K.A.J. (1967). Collembola (Springtails). Antarct. Res. Ser..

[60] Stevens M.I., Hogg I.D. (2002). Expanded distributional records of Collembola and Acari in southern Victoria Land, Antarctica. Pedobiologia.

[61] Stevens M.I., Frati F., McGaughran A., Spinsanti G., Hogg I.D. (2007). Phylogeographic structure suggests multiple glacial refugia in northern Victoria Land for the endemic Antarctic springtail *Desoria klovstadi*, (Collembola, Isotomidae). Zool. Scripta.

[62] Fanciulli P.P., Summa D., Dallai R., Frati F. (2001). High levels of genetic variability and population differentiation in *Gressittacantha terranova* (Collembola, Hexapoda) from Victoria Land, Antarctica. Antarct. Sci..

[63] Hawes T.C., Torricelli G., Stevens M.I. (2010). Haplotype diversity in the Antarctic springtail *Gressittacantha terranova* at fine spatial scales—A Holocene twist to a Pliocene tale. Antarct. Sci..

[64] Torricelli G., Frati F., Convey P., Telford M., Carapelli A. (2010). Population structure of *Friesea grisea* (Collembola, Neanuridae) in the Antarctic Peninsula and Victoria Land: evidence for local genetic differentiation of pre-Pleistocene origin. Antarct. Sci..

[65] Nolan L., Hogg I.D., Stevens M.I., Haase M. (2006). Fine scale distribution of mtDNA haplotypes for the springtail *Gomphiocephalus hodgsoni* (Collembola) corresponds to an ancient shoreline in Taylor Valley, continental Antarctica. Polar Biol..

[66] Stevens M.I., Hogg I.D. (2006). Contrasting levels of mitochondrial DNA variability between mites (Penthalodidae) and springtails (Hypogastruridae) from the Trans-Antarctic Mountains suggest long-term effects of glaciation and life history on substitution rates, and speciation processes. Soil Biol. Biochem..

[67] Sinclair B.J., Sjursen H. (2001). Cold tolerance of the Antarctic springtail *Gomphiocephalus hodgsoni* (Collembola, Hypogastruridae). Antarct. Sci..

[68] McGaughran A., Convey P., Redding G.P., Stevens M.I. (2009). Temporal and spatial metabolic rate variation in the Antarctic springtail *Gomphiocephalus hodgsoni*. J. Insect Physiol..

[69] McGaughran A., Redding G.P., Stevens M.I., Convey P. (2010). Patterns of temporal and spatial metabolic rate variation in an Antarctic springtail. J. Insect Phys..

[70] Hogg I.D., Hebert P.D.N. (2004). Biological identification of springtails (Hexapoda: Collembola) from the Canadian Arctic, using mitochondrial DNA barcodes. Can. J. Zool..

[71] Dabert J., Ehrnsberger R., Dabert M. (2008). *Glaucalges tytonis* sp. nov. (Analgoidea: Xolalgidae) from the barn owl *Tyto alba* (Strigiformes: Tytonidae): Compiling morphology with DNA barcode data for taxa descriptions in mites (Acari). Zootaxa.

[72] Porco D., Rougerie R., Deharveng L., Hebert P. (2010). Coupling non-destructive DNA extraction and voucher retrieval for small soft-bodied Arthropods in a high throughput context: the example of Collembola. Mol. Ecol. Resour..

[73] Demetras N.J., Hogg I.D., Banks J.C., Adams B.J. (2010). Latitudinal distribution and mitochondrial DNA (COI) variability of *Stereotydeus* spp. (Acari: Prostigmata) in Victoria Land and the central Transantarctic Mountains. Antarct. Sci..

[74] Courtright E.M., Wall D.H., Virginia R.A., Frisse L.M., Vida J.T., Thomas W.K. (2000). Nuclear and mitochondrial DNA sequence diversity in the Antarctic nematode *Scottnema lindsayae*. J. Nematol..

[75] Nkem J.N., Wall D.H., Virginia R.A., Barrett J.E., Broos E.J., Porazinska D.L., Adams B.J. (2006). Wind dispersal of soil invertebrates in the McMurdo Dry Valleys, Antarctica. Polar Biol..

[76] Maslen N.R., Convey P. (2006). Nematode diversity and distribution in the southern maritime Antarctic: clues to history?. Soil Biol. Biochem..

[77] Skotnicki M.L., Ninham J.A., Selkirk P.M. (2000). Genetic diversity, mutagenesis and dispersal of Antarctic mosses - a review of progress with molecular studies. Antarct. Sci..

[78] Skotnicki M.L., MacKenzie A.M., Clements M.A., Selkirk P.M. (2005). DNA sequencing and genetic diversity of the 18S–26S nuclear ribosomal internal transcribed spacers (ITS) in nine Antarctic moss species. Antarct. Sci..

[79] Romeike J., Friedl T., Helms G., Ott S. (2002). Genetic diversity of algal and fungal partners in four species of *Umbilicaria* (Lichenized Ascomycetes) along a transect of the Antarctic Peninsula. Mol. Biol. Evol..

[80] Sohlenius B., Boström S. (2006). Patch-dynamics and population structure of nematodes and tardigrades on Antarctic nunataks. Eur. J. Soil Biol..

[81] Janko K., Lecointre G., DeVries A., Couloux A., Cruaud C., Marshall C. (2007). Did glacial advances during the Pleistocene influence differently the demographic histories of benthic and pelagic Antarctic shelf fishes?—Inferences from intraspecific mitochondrial and nuclear DNA sequence diversity. BMC Evol. Biol..

[82] Holdregger R., Stehlik I., Lewis-Smith R.I., Abbott R.J. (2003). Populations of Antarctic hairgrass (*Deschampsia antarctica*) show low genetic diversity. Arctic Antarct. Alpine Res..

[83] Allegrucci G., Carchine G., Todisco V., Convey P., Sbordoni V. (2006). A molecular phylogeny of Antarctic chironomidae and its implications for biogeographical history. Polar Biol..

[84] Sugden D.E., Bentley M.J., Cofaigh C.O. (2006). Geological and geomorphological insights into Antarctic ice sheet evolution. Philos. T. Roy. Soc. Lond. A.

[85] Convey P., Domack E., Burnett A., Leventer A., Convey P., Kirby M., Bindschadler R. (2003). Maritime Antarctic climate change: signals from terrestrial biology. Antarctic Peninsula Climate Variability: Historical and Palaeoenvironmental Perspectives. Antarctic Research Series.

[86] Chown S.L., Hull B., Gaston K.J. (2005). Human impacts, energy availability and invasion across Southern Ocean Islands. Global Ecol. Biogeogr..

[87] Chown S.L., Davies S., Joubert L. (2006). Prince Edward Islands Environmental Management Plan Version 0.1..

[88] Nougier J., Adie R.J. (1972). Geochronology of the volcanic activity in Îles Kerguelen. Antarctic Geology and Geophysics.

[89] Mercer J.H. (1983). Cenozoic glaciation in the southern hemisphere. Annu. Rev. Earth Pl. Sc..

[90] Hall K.J. (1990). Quaternary glaciations in the southern ocean: sector 0° long–180° long. Quaternary Sci. Rev..

[91] Hall K.J. (2002). Review of present and quaternary periglacial processes and landforms of the maritime and sub-Antarctic region. S. Afr. J. Sci..

[92] McDougall I., Verwoerd W., Chevallier L. (2001). K-Ar geochronology of Marion Island, Southern Ocean. Geol. Mag..

[93] Ruddell A., Green K., Woehler E. (2006). An inventory of present glaciers on Heard Island and their historical variation. Heard Island: Southern Ocean Sentinel.

[94] Grobler G.C., Janse van Rensburg L., Bastos A.D.S., Chimimba C.T., Chown S.L. (2006). Molecular and morphometric assessment of *Ectemnorhinus* weevil species (Coleoptera, Curculionidae, Brachycerinae) from the sub-Antarctic Prince Edward Islands. J. Zool. Syst. Evol. Res..

[95] McGaughran A., Stevens M.I., Holland B.R. (2010). Biogeography of circum-Antarctic springtails. Mol. Phylogenet. Evol..

[96] Myburgh M., Chown S.L., Daniels S.R., Jansen van Vuuren B. (2007). Population structure, propagule pressure, and conservation biogeography in the sub-Antarctic: Lessons from indigenous and invasive springtails. Divers. Distrib..

[97] Marshall D.J., Convey P. (2004). Latitudinal variation in habitat specificity of ameronothrid mites (Oribatida). Exp. Appl. Acarol..

[98] Wallwork J.A. (1973). Zoogeography of some terrestrial microarthropoda in Antarctica. Biol. Rev..

[99] Hawes T.C., Worland M.R., Bale J.S., Convey P. (2008). Rafting in Antarctic Collembola. J. Zool..

[100] McGaughran A., Hogg I.D., Convey P. Antarctic terrestrial biodiversity and potential ecophysiological responses to climate change: A springtail (Collembola) perspective. Polar Biol..

[101] McGaughran A., Convey P., Stevens M.I., Chown S.L. (2010). Metabolic rate, genetic and microclimate variation among springtail populations from sub-Antarctic Marion Island. Polar Biol..

[102] Mortimer E., Jansen van Vuuren B. (2007). Phylogeography of *Eupodes minutus* (Acari: Prostigmata) on sub-Antarctic Marion Island reflects the impact of historical events. Polar Biol..

[103] Cowan D.A., Pointing S.B., Stevens M.I., Cary S.C., Stomeo F., Tuffin I.M. (2011). Distribution and abiotic influences on hypolithic microbial communities in an Antarctic Dry Valley. Polar Biol..

[104] SCAR Antarctic Biodiversity database. http://data.aad.gov.au/aadc/biodiversity/.

[105] Antarctic Digital Database version 5 © Scientific Committee on Antarctic Research 1993-2006. http://www.add.scar.org:8080/add/.

[106] Edwards S.V., Beerli P. (2000). Perspective: Gene divergence, population divergence, and the variance in coalescence time in phylogeographic studies. Evolution.

[107] Hare M.P. (2001). Prospects for nuclear gene phylogeography. Trends Ecol. Evol..

[108] Templeton A.R. (2002). Out of Africa again and again. Nature.

[109] Garrick R.C., Rowell D.M., Simmons C.S., Hillis D.M., Sunnucks P. (2008). Fine-scale phylogeographic congruence despite demographic incongruence in two low-mobility saproxylic springtails. Evolution.

